# Failure Mode Classification for Rolling Element Bearings Using Time-Domain Transformer-Based Encoder

**DOI:** 10.3390/s24123953

**Published:** 2024-06-18

**Authors:** Minh Tri Vu, Motoaki Hiraga, Nanako Miura, Arata Masuda

**Affiliations:** 1 Division of Mechanophysics, Graduate School of Science and Technology, Kyoto Institute of Technology, Kyoto 606-8585, Japan; m2623501@edu.kit.ac.jp; 2 Faculty of Mechanical Engineering, Kyoto Institute of Technology, Kyoto 606-8585, Japan; hiraga@kit.ac.jp (M.H.); miura-n@kit.ac.jp (N.M.)

**Keywords:** failure mode classification, smart diagnostics, vibrations, signal denoising, failure detection

## Abstract

In this paper, we propose a Transformer-based encoder architecture integrated with an unsupervised denoising method to learn meaningful and sparse representations of vibration signals without the need for data transformation or pre-trained data. Existing Transformer models often require transformed data or extensive computational resources, limiting their practical adoption. We propose a simple yet competitive modification of the Transformer model, integrating a trainable noise reduction method specifically tailored for failure mode classification using vibration data directly in the time domain without converting them into other domains or images. Furthermore, we present the key architectural components and algorithms underlying our model, emphasizing interpretability and trustworthiness. Our model is trained and validated using two benchmark datasets: the IMS dataset (four failure modes) and the CWRU dataset (four and ten failure modes). Notably, our model performs competitively, especially when using an unbalanced test set and a lightweight architecture.

## 1. Introduction

Fault diagnosis, i.e., the classification of variables of interest at multiple fault detection, abnormal detection, or fault classification, is a crucial problem in many industrial systems, especially in rotating machines. Faults that occur in rotating components such as gears and bearings may cause subsequent severe damage or even the breakdown of the whole machine. Vibration signal-based fault detection has been intensely investigated as the most common approach to machine diagnosis, and recent studies have paid growing attention to the application of machine learning.

The practical classification of faults has proven to be most successful when employing data-driven approaches, as demonstrated by various studies [1,2]. Deep neural networks (DNNs) have emerged as a prominent branch within machine learning and have seen increasing adoption in the fault diagnosis of rotating machinery [3]. A common approach involves developing a feature extractor to transform vibration signals into a feature space, with the extracted features serving as input for DNNs. For instance, Xu et al. [4] proposed a fault diagnosis method for rolling bearings using vibration signals, employing variational mode decomposition for preprocessing and deep convolutional neural networks (DCNNs) for fault classification. Michau et al. [5] introduced an approach based on hierarchical extreme learning machines (HELMs), which utilized an autoencoder for unsupervised feature learning. Li et al. [6] presented a convolutional neural network model integrated with a wavelet filter for mechanical vibration signals. Additionally, Magar et al. [7] introduced FaultNet, a deep convolutional neural network that combined various signal processing techniques and machine learning techniques for feature extraction and fault classification. Tan et al. [8] introduced fault classification methods employing sequential forward selection to obtain features from vibration datasets, followed by training an integrated model comprising an extreme learning machine and logistic mapping. Other approaches focus on feature decomposition, with an emphasis on effective feature extraction. For example, Wang et al. [9] proposed a framework integrating singular spectrum decomposition (SSD) to generate practical spectral components, along with a neural network configured by a stochastic configuration network, which was also utilized in [10]. Li et al. [11] developed data augmentation methods called variational mode reconstruction (VMR) to enrich training data, with the augmented dataset subsequently used to train a deep residual shrinkage network.

Several architectures based on variants of recurrent neural networks (RNNs) have also been explored within the context of fault diagnosis. Liu et al. [12] utilized a stacked RNN and autoencoder for feature extraction in bearing datasets. Similarly, Zhao et al. [13] introduced a similar approach using an LSTM neural network to process chemical process data directly and diagnose fault types.

However, in DNN- and RNN-based models, the architectures are often treated as black boxes, where model predictions are influenced by numerous parameters and their complex nonlinear interactions. This can lead to poor explanatory quality. Moreover, a significant drawback of these models is their limited capacity to capture multiscale correlations inherent in fault signals, ranging from microscale vibration dynamics to longer timescales associated with failure scenarios.

Recently, attention mechanism-based models with inherent interpretability have garnered attention, primarily in the field of natural language processing (NLP). Specifically, the Transformer [14], which features fully self-attention mechanisms and does not rely on recursion or convolution, has outperformed past approaches like RNNs on machine translation tasks in terms of translation quality and training cost. Furthermore, its successor technologies such as BERT and GPT have achieved great success in various NLP tasks and revolutionized natural language-based AI technologies.

A few approaches with Transformer-based architectures have been demonstrated in the machine diagnosis field and have shown successful performance. Ding et al. [3] utilized the wavelet transform to convert time domain data into the time-frequency domain, then trained them with a pure Transformer encoder. Zhou et al. [15] employed stacked convolutional layers for preprocessing and then adopted a similar approach by using a vanilla Transformer encoder. In [16], “images” were created as two-dimensional data matrices generated from continuous wavelet transform (CWT), which were then processed by an elaborate Transformer vision model. The same methodology was applied in [17], where a symmetric dot pattern (SDP) transformation method was used to convert vibration signals into two-dimensional images, followed by training multiscale convolutional layers combined with a Transformer encoder on the converted images.

Many current Transformer-based architectures incorporate additional preprocessing methodologies alongside the Transformer. However, the self-attention mechanism inherent in the Transformer should theoretically be capable of capturing the heterogeneous structure of vibration signals and providing meaningful time-ordering features on its own, similar to its success in the field of NLP. Specifically, the stacked architecture of Transformer encoders, each consisting of multi-head self-attention layers, enables the modeling of failure signals from rotating machinery by considering both local correlations over multiple time steps and global temporal interactions. This is particularly crucial for identifying recurring patterns embedded in the failure signals.

In this study, we introduce a Transformer encoder-based network for diagnosing faults in rolling element bearings and validate its performance using benchmark datasets. Our paper contributes in three main ways: Firstly, we propose the integration of a lightweight and real-time efficient denoising method with a Transformer model, which enhances data quality prior to training without necessitating costly and explicit data transformation methods. Secondly, to leverage the feature extraction capabilities of the Transformer encoder architecture, we directly input raw data (in the time domain) into a custom Transformer encoder. This simplifies the original architecture by eliminating the need for preprocessing layers, except for data cleaning, demonstrating that, with a simple and appropriate design, effective learning from raw data can still be achieved. Additionally, we validate our method using two benchmark datasets. The results indicate that our proposed framework achieves accurate predictions comparable to other machine learning methods, despite its simple network design. Lastly, we provide comprehensive details about all utilized algorithms, data analysis, and a complete metric evaluation for a classification task, enhancing readers’ understanding of our framework and results.

The remainder of this paper is structured as follows: Section 3 introduces our methodology and provides a detailed description of the architecture of the proposed denoising Transformer-based framework. In Section 4, we outline the characteristics of the two datasets, conduct data analysis, and compare our approach to related works that have utilized the same datasets. Finally, Section 5 concludes the paper.

## 2. Related Works

To understand the underlying patterns in vibration data, many research works have utilized transformation methods to convert 1D vibration signal data into images, which are suitable for applying deep learning techniques. For instance, Mao et al. [18] employed the fast Fourier transform (FFT) to process input signals into a spectrum data form. The transformed spectrum was then sampled by a generative adversarial network (GAN) to produce artificial samples for the minority defect by adding random noise. Xu et al. [19] transformed 1D vibration signals into 2D time-frequency spectra with abundant condition information using continuous Morlet wavelet transforms. The 2D spectrum data, represented as grayscale images, were then fed into a convolutional neural network model based on LeNet-5, which served as the encoder. The encoded features were trained using a random forest (RF) model for fault diagnosis. Interestingly, Du et al. [20] proposed the integrated gradient-based continuous wave transform (IG-CWT) method, where a signal is converted into time-frequency images after performing two CWTs. The first CWT generates sample images for the IG module to grade the important frequency components, which serve as inputs for the second CWT. Although these methods have achieved good performance, they still have some drawbacks, such as a lack of adaptability or problem-specific setup. For example, choosing FFT parameters and mother wavelet functions is crucial but often only suitable for specific datasets.

Considering that deep learning models can directly work on raw data without any preprocessing, several studies have focused on bearing fault diagnosis using deep learning directly on raw vibration data. Yuan et al. [21] developed a system that automatically extracts hidden degradation features from noisy, time-series data before feeding the transformed data into a CNN model. The CNN model’s predictions are then fed back into the machinery model to identify failure types. Due to the inherent complexity of the hidden layers, understanding how the learned model works can be challenging. The data samples are selected and reconstructed from the original data, with each sample having the same time course. Li et al. [22] introduced a technique for learning deep distance metrics using a deep CNN as the dominant architecture. The method increases the length of inter-class differences while minimizing the distance between intra-class variations through a representation clustering technique. A domain adaptation method is also adopted to reduce the maximum mean discrepancy between training and testing data. However, achieving 99.34% model accuracy requires a sample length of 8192, and the training process takes about 40 min on average. Wang et al. [23] proposed a reinforcement neural architecture search method, which includes two models: a controller based on reinforcement learning and a child model based on CNN. The controller, acting as an agent, creates a set of hyperparameters as the agent’s action to build the CNN architecture and uses the accuracy of the child models as the reward. This approach can be seen as a Markov decision process, with the CNN architecture being discovered by maximizing the reward. They adjusted the parameters using the policy gradient method since the reward is not differentiable. However, the number of viable options for building child models is vast, and the research scope is broad. Despite generating random actions to prevent local optima, it is still easy to get stuck in local optimal solutions.

Based on the above analysis, it is clear that the key to achieving a low-cost, adaptable model lies in effectively exploiting the underlying patterns from raw data without hard-coding for specific problems. Here, for the first time, we train an attention-based model directly on the vibration data and evaluate the model’s performance using diverse metrics. We provide a comprehensive failure mode diagnosis that demonstrates superior performance compared to many of the established approaches.

## 3. Method

### 3.1. Data Cleaning and Denoising

To enhance the quality of the dataset and enable machine learning models to effectively identify useful patterns and features, the raw vibration data underwent preprocessing steps. Initially, the data were detrended using the standard detrend function in the Python (3.8.2) library SciPy (1.9.1) to remove linear trend because the data had a long-term trend that was mostly an offset. The effect of detrending is later demonstrated in Section 4.1.2. Subsequently, each sample of the detrended dataset underwent denoising using an algorithm proposed by [24]. This denoising algorithm, known as DeSpaWN, is an unsupervised neural network inspired by wavelet denoising techniques. DeSpaWN utilizes learnable filter coefficients and threshold biases to minimize a specific loss function, which comprises the sum of the *ℓ*_1_ norm of the reconstruction residual and the *ℓ*_1_ norm of the wavelet coefficients. By optimizing this loss function, DeSpaWN achieves optimal wavelet denoising, effectively minimizing reconstruction error while maximizing sparsity in the wavelet coefficients. The algorithm integrates convolutional kernels and thresholding mechanisms, akin to conventional wavelet denoising, but with the added flexibility and adaptability afforded by the learnable parameters.

### 3.2. Transformer Model

The Transformer architecture, initially developed for natural language processing (NLP), incorporates a self-attention mechanism that enables the modeling of multiscale relationships within text, spanning from word-to-word interactions to broader paragraph-level context. This modeling capability, intrinsic to the Transformer, holds promise for fault analysis and prediction tasks, where capturing multiscale correlations within failure signals is crucial.

At its core, the original Transformer architecture comprises several key components. The first of these is token embedding, which serves as a mechanism for representing characters or vocabulary elements in vector form. When applied to time-series data, each data sequence is segmented into short segments, referred to hereafter as “data tokens”, with a length of *d*. These data tokens are then directly input into the network. The process of tokenizing the data involves reshaping the data sequence into a matrix $X={\left[x_{1}^{T},\ldots ,x_{l_{x}}^{T}\right]}^{T}\in R^{l_{x}\times d}$, where $x_{n}$ represents a row vector corresponding to the *n*th token, and $l_{x}$ denotes the total number of tokens in the data sequence.

**Figure 1:**
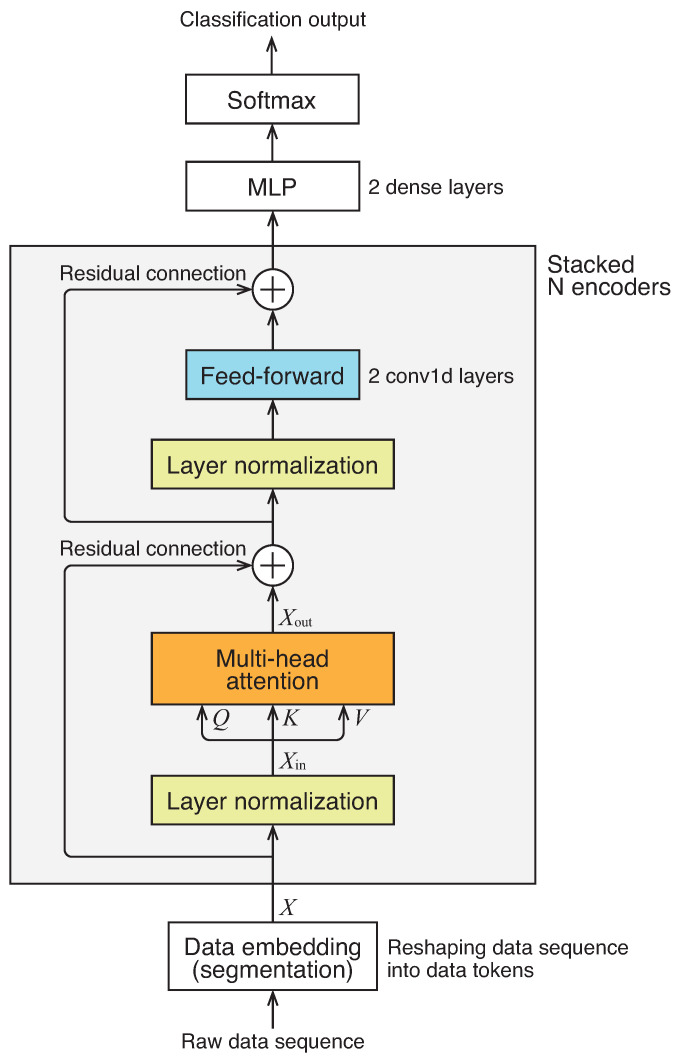
Architecture of network.

The overall architecture of the network we employed is illustrated in Figure 1. It consists of a series of *N* encoder networks followed by multi-layer perceptrons to generate a classification output. It is worth noting that, unlike the original Transformer, we do not utilize positional encoding or masking.

The encoder, highlighted by the boxed section in the figure, comprises several components: a multi-head self-attention block, layer normalization, feed-forward networks, and residual connections. These components closely resemble those of the original Transformer encoder, albeit with a minor modification in the connection order. The multi-head self-attention mechanism, a central element of the architecture, enables the model to learn contextual information within the input data, capturing the “mutual relationship” among all data tokens in the sequence. It transforms the input data matrix $X_{\mathrm{in}}$ into an output data matrix of the same size, denoted as $X_{\mathrm{out}}$, through the following equations:

(1) $$X_{\mathrm{out}}=\mathrm{MultiHeadSelfAttention}\left(X_{\mathrm{in}}\right)=\left[H_{1},\ldots ,H_{h}\right]W^{O}$$

where $W^{O}\in R^{hd_{v}\times d}$ is a trainable matrix, and $H_{i}\in R^{l_{x}\times d_{v}}$ is the *i*th head calculated as

(2) $$H_{i}=\mathrm{Attention}\left(X_{\mathrm{in}}W_{i}^{Q},X_{\mathrm{in}}W_{i}^{K},X_{\mathrm{in}}W_{i}^{V}\right)$$

where Attention stands for the scaled dot-product attention defined as

(3) $$\mathrm{Attention}\left(Q,K,V\right)=\mathrm{Softmax}\left(\frac{QK^{T}}{\sqrt{d_{k}}}\right)V$$

where Softmax stands for the softmax function defined as $\mathrm{Softmax}\left(x_{i}\right)=\exp\left(x_{i}\right)/\sum_{j}\exp\left(x_{j}\right)$ applied to each row of the argument matrix, and *Q*, *K*, and *V* are matrices defined as $Q={\left[q_{1}^{T},\ldots ,q_{l_{x}}^{T}\right]}^{T}$, $K={\left[k_{1}^{T},\ldots ,k_{l_{x}}^{T}\right]}^{T}$, and $V={\left[v_{1}^{T},\ldots ,v_{l_{x}}^{T}\right]}^{T}$, where $q_{n}\in R^{d_{k}}$, $k_{n}\in R^{d_{k}}$, and $v_{n}\in R^{d_{v}}$ are row vectors referred to as query, key, and value, respectively, and $d_{k}$ is the key vector’s dimension and $d_{v}$ is the value vector’s dimension. The matrices $W_{i}^{Q}\in R^{d\times d_{k}}$, $W_{i}^{K}\in R^{d\times d_{k}}$, and $W_{i}^{V}\in R^{d\times d_{v}}$ in Equation (2) are trainable matrices that map the data tokens into query, key, and value vectors, respectively. In this work, we employ the number of heads h=4 and $d_{k}=d_{v}=d/h$.

The attention mechanism described in Equation (3) computes the average of the value vectors *V*, weighted by the similarity scores between a specific query $q_{n}$ and all keys $k_{1},\ldots ,k_{l_{x}}$. Since the queries *Q*, keys *K*, and values *V* are derived from the input $X_{\mathrm{in}}$ through linear mapping, as outlined in Equation (2), this mechanism enables the network to extract features $H_{i}$ while considering the contextual information of the input vectors $X_{\mathrm{in}}$, which represent the layer-normalized data tokens for the first encoder. Additionally, these features belong to a subspace with reduced dimensionality of d/h. By aggregating weighted collections of features from *h* different subspaces, as described in Equation (1), this multi-head network can effectively manage the contextual information of the input data from *h* different perspectives.

## 4. Validation

Two case studies using standard bearing datasets were carried out to validate the proposed algorithm.

### 4.1. Validation Using IMS Dataset

#### 4.1.1. Description of Dataset

The IMS dataset [25] is derived from a bearing test rig system. This rig comprises four bearings mounted on a shaft, with each bearing equipped with both vertical and horizontal accelerometers. Details of the experiments conducted with this dataset are outlined in Table 1. The data files were recorded at 10 min intervals, with each file containing vibration signal snapshots captured every second. Each data file contains 20,480 data points, representing a duration of 1.0 s at the 20 kHz sampling frequency of the accelerometers.

#### 4.1.2. Results and Discussions

To clearly demonstrate the performance improvement resulting from preprocessing data and leveraging the attention mechanism from the vanilla Transformer, we carried out an empirical analysis based on two primary design components:

- Attention-based model with a simplified structure: Unlike the original design proposed by Vaswani et al. [14], which comprises a full encoder–decoder architecture, we focused on utilizing only the Transformer encoder. We stacked multiple encoder blocks and integrated an MLP layer for classification.
- Implementing a robust adaptive denoising filter: Since different signals may contain varying levels of noise, employing a robust adaptive denoising filter allows for the automatic decomposition and reconstruction of raw data using learnable thresholds.

**Figure 2:**
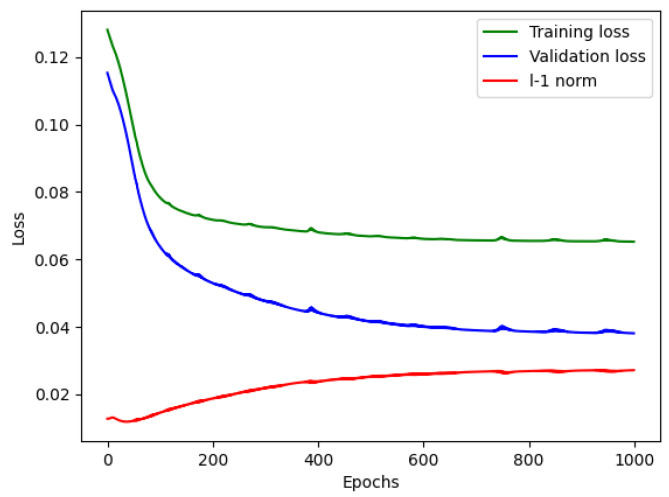
Denoising model performance.

**Figure 3:**
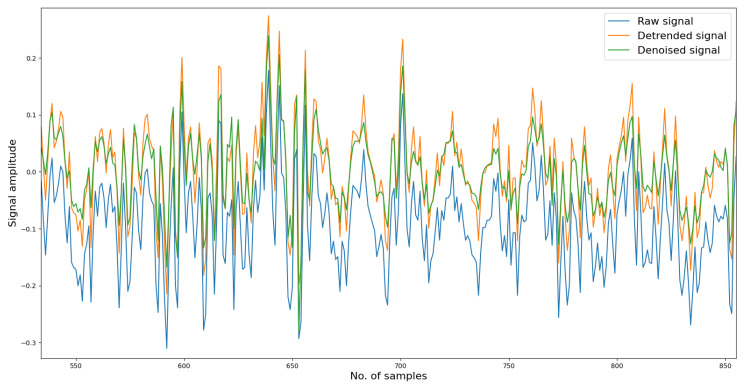
Denoised and reconstructed results from raw data.

To begin with, in the denoising phase, each vibration signal file consists of 20,480 samples. We divided these samples into 16,000 for training and 4480 for testing. Initially, the reconstructed signal may exhibit fluctuations in several samples, but as the denoising model learns more information, it gradually fits the remaining data samples, resulting in a well-reconstructed signal. We trained the denoising model for approximately one thousand epochs. As depicted in Figure 2, the reconstruction loss remains around 0.08, and the L1 norm of the reconstruction error is approximately 0.03, indicating a significant improvement in data quality, which aids in distinguishing variations in the signals. Figure 3 depicts a segment of the signal alongside its corresponding detrended and denoised results.

**Figure 4:**
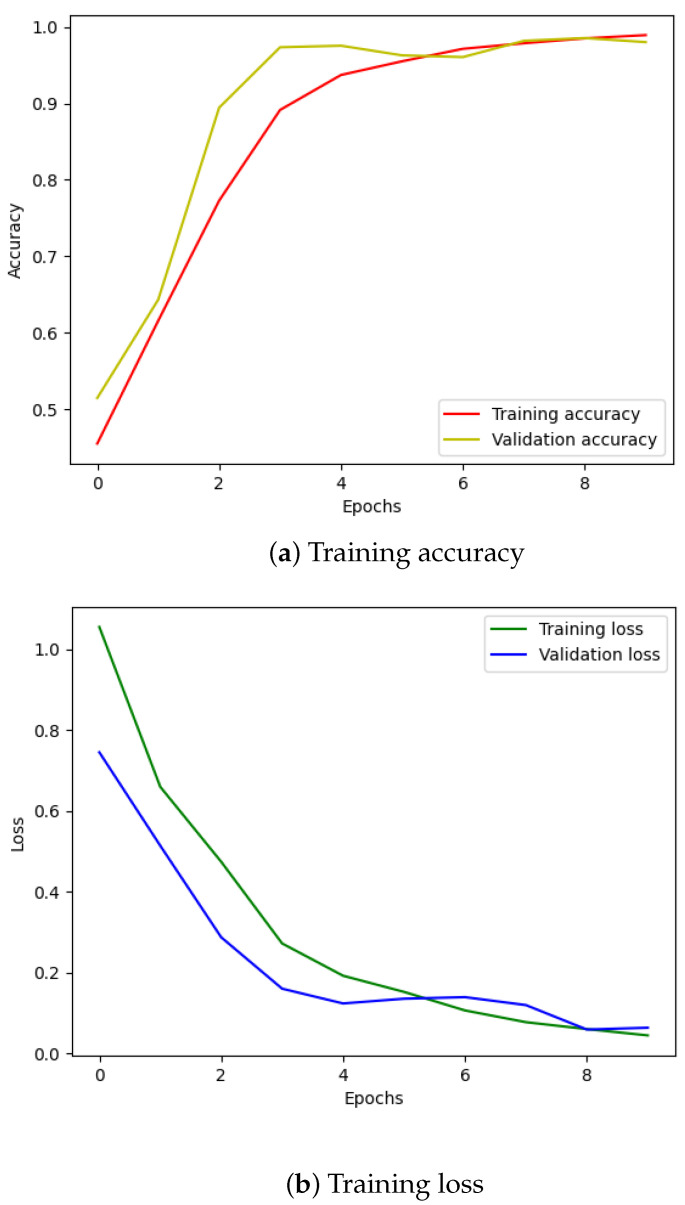
Training results using IMS dataset.

Moving on to the classification phase, we divided the denoised data files into four groups, each containing 750 files representing one of the four failure scenarios. These filtered datasets were then fed into the Transformer model for training. The training set comprised approximately 2600 files, with around 600 files allocated for testing. As illustrated in Figure 4, the accuracy improved significantly, and the loss converged within just a few epochs.

**Figure 5:**
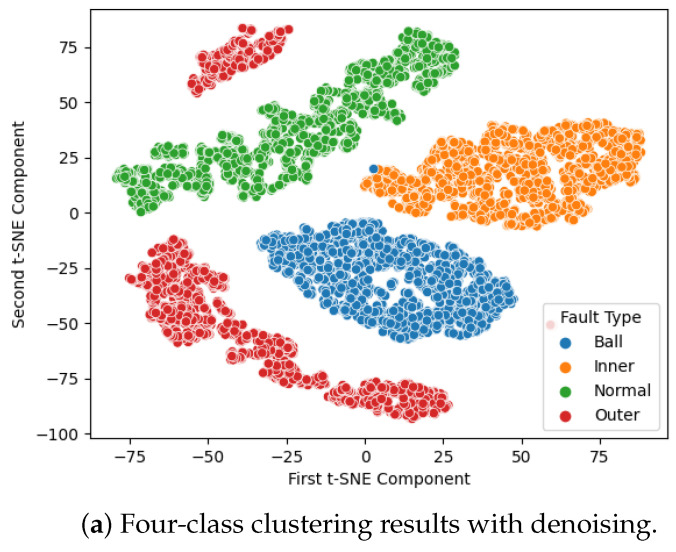

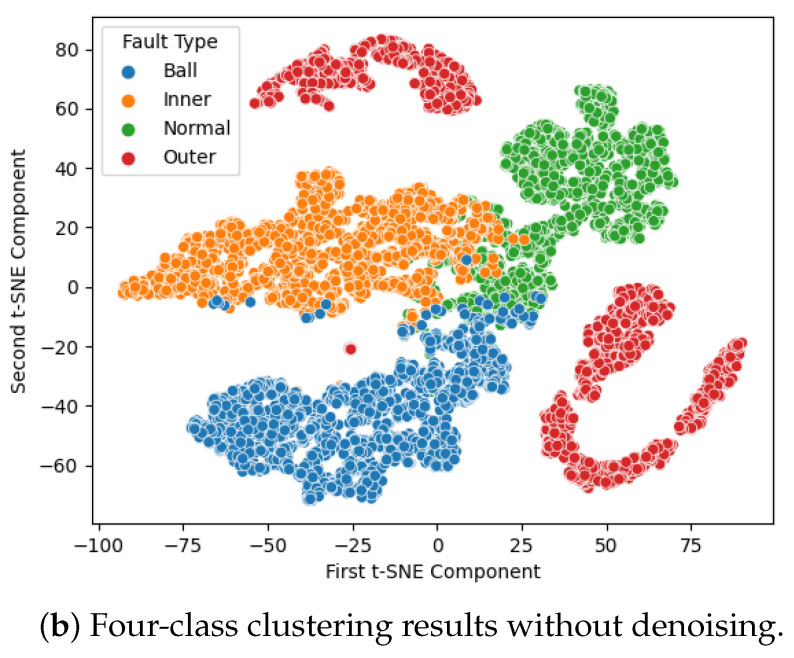
IMS dataset, 4-class clustering results.

The classification results are presented in Figure 5. In this graph, each sample point represents a label with corresponding color encoding for each failure mode. To demonstrate how effectively the model can classify each failure mode, we employ t-distributed stochastic neighbor embedding (t-SNE) for dimensionality reduction visualization, as shown in Figure 5. The clusters observed in Figure 5a indicate that the noise reduction and modified Transformer effectively project the data into separated spaces, leading to improved classification results. In contrast, without denoising, there is a significant overlap of data points in the data space, resulting in erroneous predictions.

**Figure 6:**
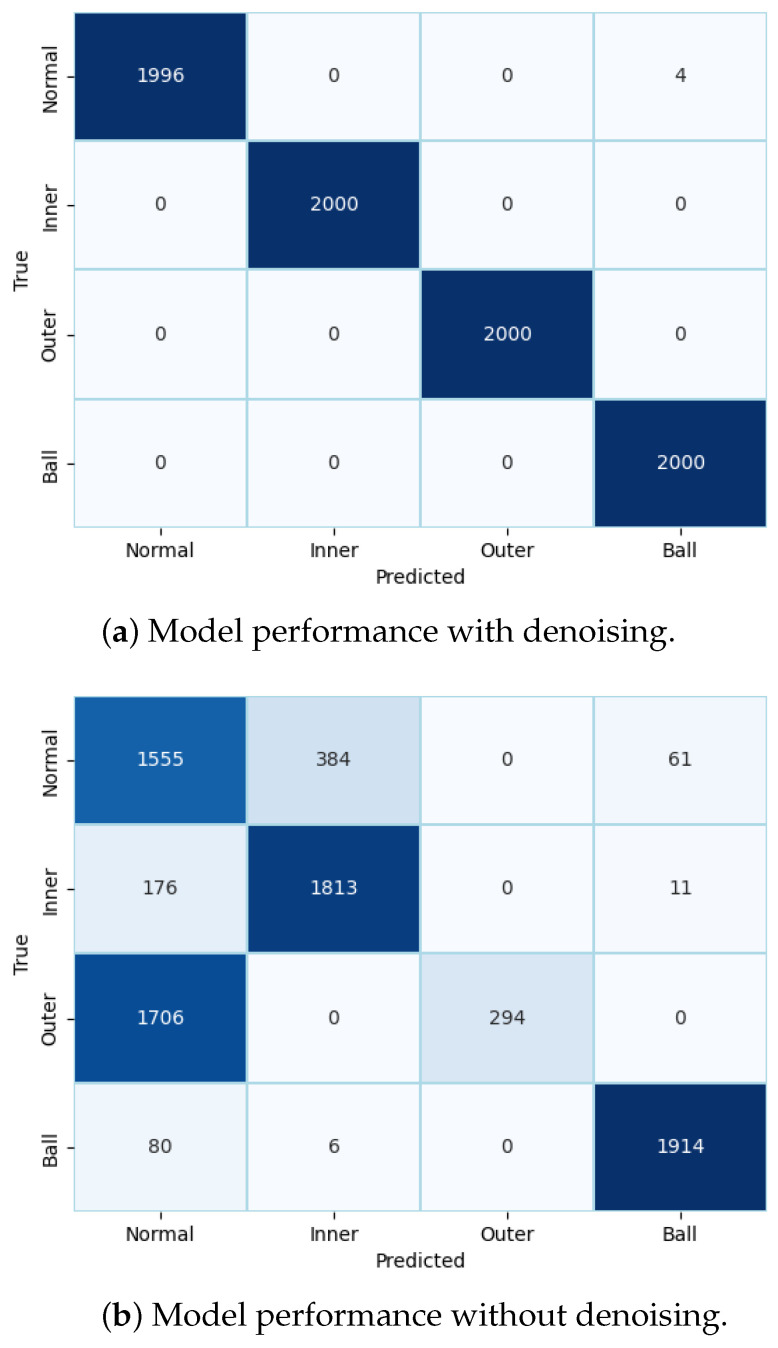
Metric evaluation for model performance using IMS dataset.

Finally, to evaluate the performance of the model, we conducted metric evaluations. Two confusion matrices are presented in Figure 6 to compare the performance of the model with and without preprocessed raw data. Figure 6a clearly demonstrates the improved classification results of the integrated model, with minimal mispredictions. In contrast, without noise reduction from the original data, the model exhibited numerous incorrect predictions.

### 4.2. Validation Using CWRU Dataset

#### 4.2.1. Dataset Description

The CWRU dataset [26], provided by Case Western Reserve University Bearing Data Center, includes measurements from healthy and unhealthy bearings obtained from base (BA), drive end (DE), and fan end (FE) positions in the system. It contributes to advancing the application of machine learning in predictive maintenance of industrial machinery, particularly in the area of data-driven fault diagnosis. One common task in this research domain is fault detection and classification, and several other studies on bearing fault diagnosis have utilized the CWRU dataset. In this graph, we utilized a subset of this comprehensive dataset, as further detailed in Table 2.

The dataset includes data representing normal conditions and three types of faults occurring in the ball, inner race, and outer race elements of the bearing. Each element has three fault diameters: 0.007, 0.014, and 0.021 inches. This results in a ten-class classification problem aimed at distinguishing between different defect diameters and a four-class classification problem focused on classifying fault locations as ball, inner race, outer race, or normal.

#### 4.2.2. Results and Discussions

In our research, we aim to determine which faults are better classified using accelerometer signals by conducting exploratory data analysis (EDA) on the dataset. We calculate nine features for this analysis: maximum, minimum, mean, standard deviation, root mean square (RMS), skewness, kurtosis, crest factor, and form factor. These features help uncover insights into the data, allowing us to focus on preprocessing steps such as enrichment, denoising, and balancing, particularly for faults that require special attention. Each feature is computed for time segments consisting of 2048 points, equivalent to 0.04 s at the 48 kHz accelerometer sampling frequency.

**Figure 7:**
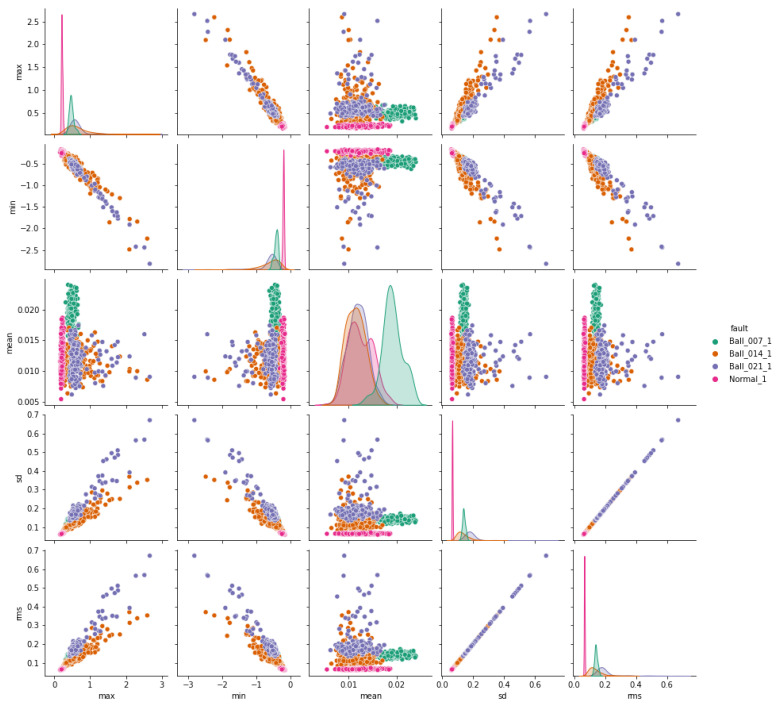
Ball vs. normal bearing features correlation.

**Figure 8:**
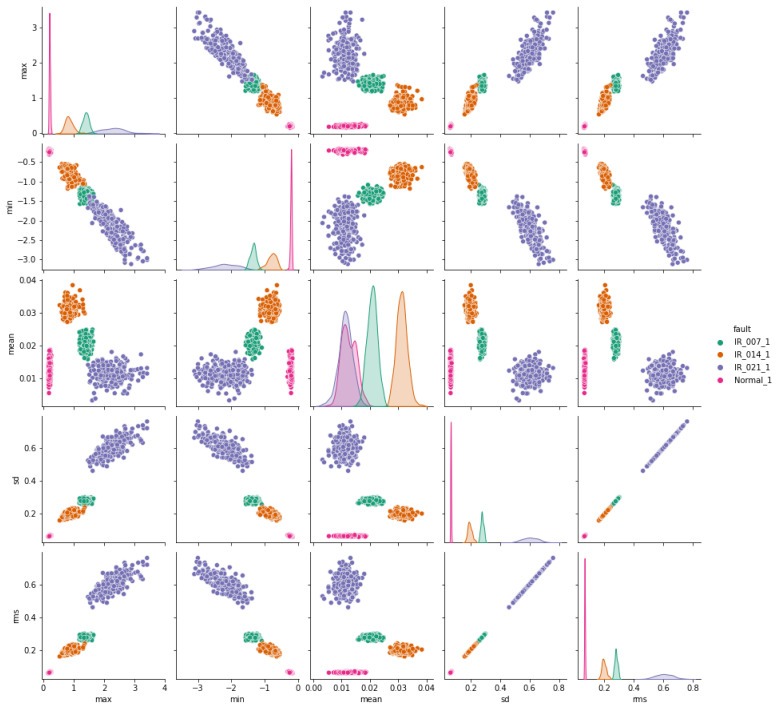
Inner-race vs. normal bearing features correlation.

**Figure 9:**
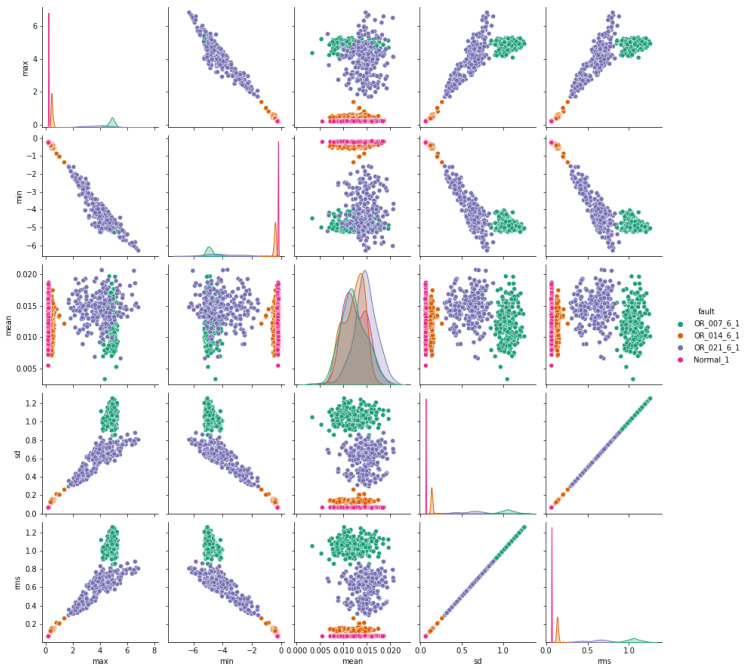
Outer-race vs. normal bearing features correlation.

The pair plots depicted in Figure 7, Figure 8 and Figure 9 show correlation matrices between normal operation and each failure mode, highlighting the distinct characteristics of bearings in each failure mode and their differences compared to those of a healthy bearing and each defect diameter size. Figure 9 specifically emphasizes the complexity of outer race defects.

For classification training, the failure data vary across different modes. We allocate 10% of all files for validation, 80% for training, and the remaining 20% for testing purposes.

**Figure 10:**
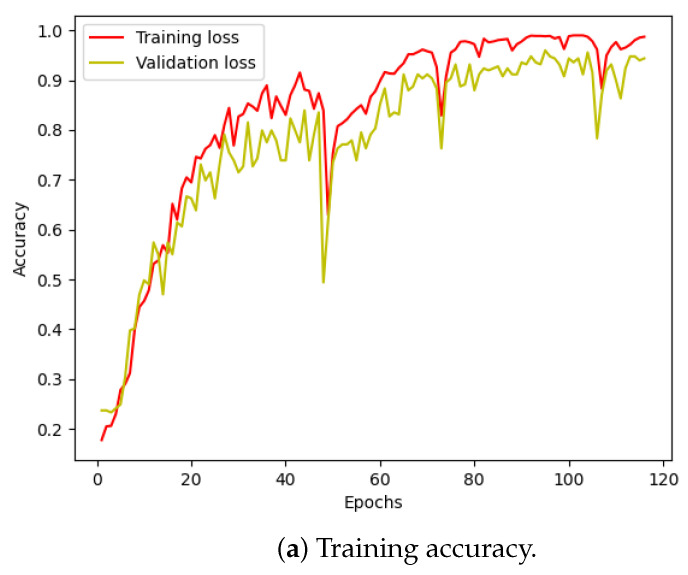

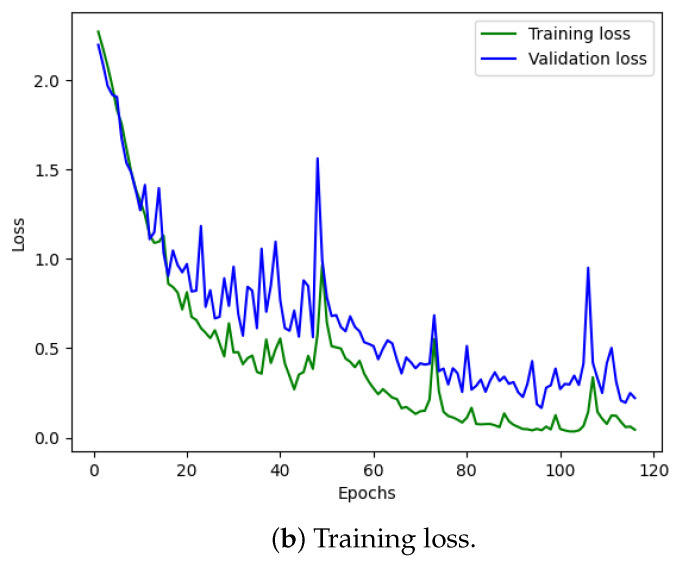
CWRU dataset, model training performance with 10 classes.

**Figure 11:**
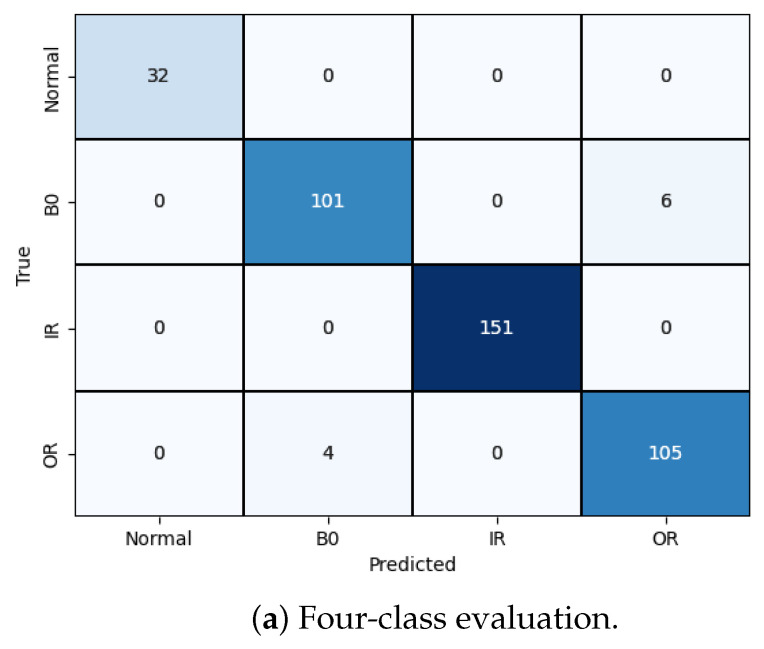

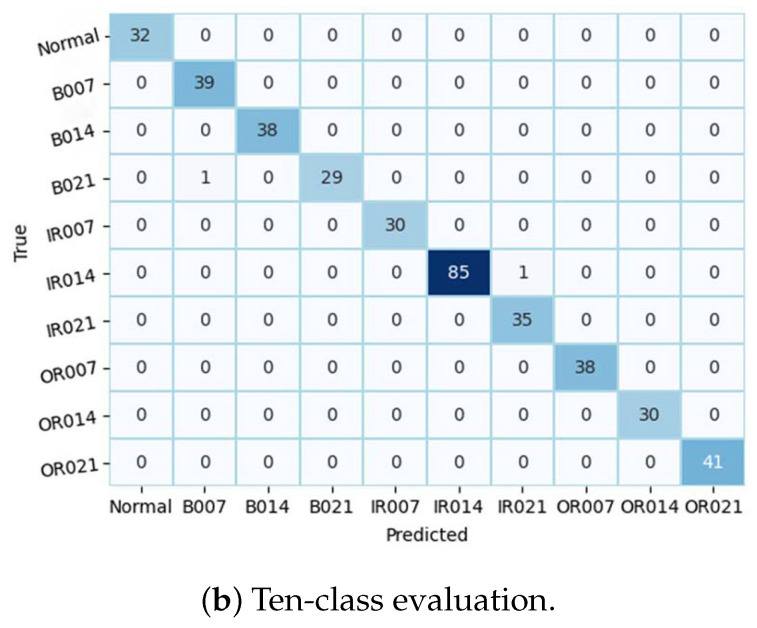
Model validation performance.

Figure 10 illustrates the training and validation (accuracy, loss) of the best model in the ten-class classification case. The accuracy and loss indicators exhibit some fluctuation. We employed early stopping with a patience of 10, which means that if the accuracy value does not improve after 10 training epochs, the training will finish and save the best model. The best accuracy of the model is approximately 99% for the training set and 95% for the validation set. To evaluate the model’s performance, we used the best saved model to make predictions on the test set, and the results, presented in a confusion matrix, are shown in Figure 11. The labels ‘B007’, ‘OR014’, and ‘IR021’ denote ball-fault bearings with a diameter of 0.007 inches, outer-race faults with a diameter of 0.014 inches, and inner-race faults with a diameter of 0.021 inches, respectively. Despite the ten classes to classify and the varying number of data samples in each class, the model performed well and achieved around 99.5% accuracy on the test set. Table 3 displays the classification report of the best model for the 10-class classification.

**Figure 12:**
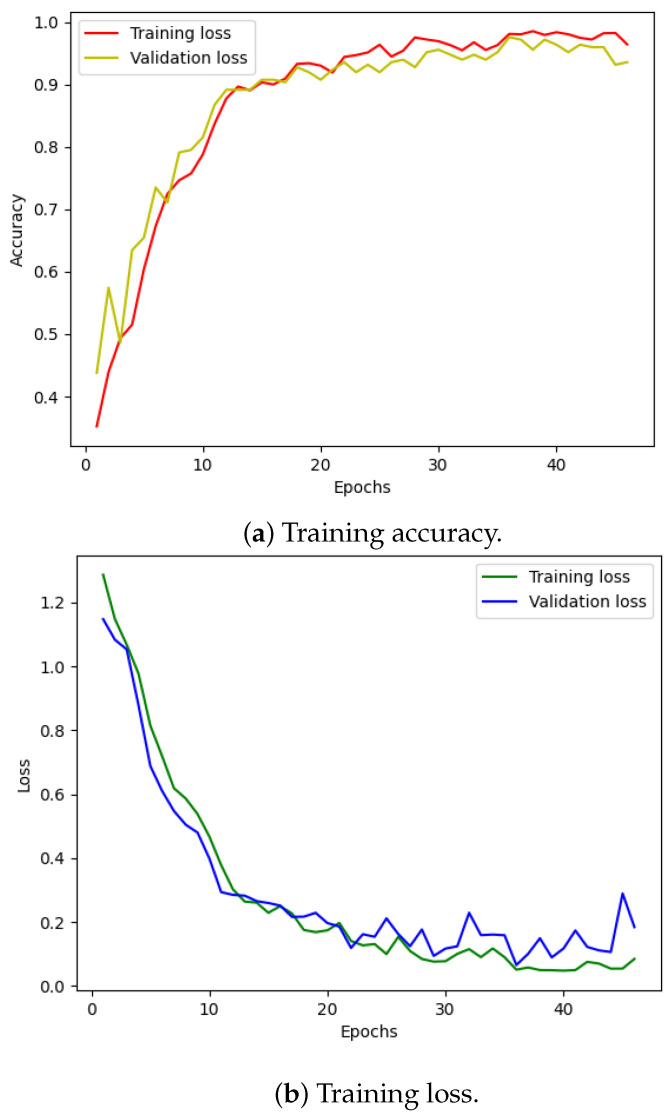
CWRU dataset, model training performance with 4 classes.

We followed a similar procedure for the four-class classification problem. Figure 12 displays the training loss and accuracy of our model. We observed that our model achieved the best accuracy and minimum loss after approximately 50 epochs. The results for the four-class classification are visualized in Figure 11a, and the corresponding classification report is presented in Table 4. Despite training with unbalanced data, our model effectively classified each failure mode, with only a minor incidence of incorrect classification.

### 4.3. Efficiency vs. Accuracy

There are several limitations in previous research, which can be categorized into two main factors. First, when converting 1D data into 2D images, such as using image representations, it can enhance classification performance, but it heavily depends on the length of the input data and the size of the converted image. Each dataset may require a specific image size to achieve good results, and noise can affect the outcome if the preprocessing step does not effectively denoise the data. Additionally, when employing wavelet transform, the choice of wavelet family introduces variability, with each option potentially being more suitable for a particular dataset. Moreover, the average training time can be considerable. For example, in [22], although the accuracy is high, the training time is 40 min, which is time-consuming. Comparison results with the previous works that have employed machine learning approaches on the same CWRU bearing dataset are provided in Table 5. Due to varying testing methods, we only report the best accuracy. In addition to that, we include other metrics such as precision, recall, and F1-score, which are the most suitable metrics for classification tasks, with the results also presented in Table 3 and Table 4. Another limitation is that the sample length is fixed, and each fault data sample has the same number of data points. While researchers can preprocess data by grouping it into a balanced dataset with consistent features from a large historical dataset, in practice, it can be challenging to create a balanced dataset with purely representative features. To address the aforementioned challenges, our approach involves using raw data directly from the dataset without balancing it and without converting it into images. The training time is remarkably efficient.

## 5. Conclusions and Future Works

In this paper, we propose a Transformer-based encoder architecture integrated with an unsupervised denoising method to learn meaningful and sparse representations of vibration signals without the need for data transformation or pre-trained data. Our architecture achieves accurate results for failure mode classification comparable to other machine learning methods despite its simple network design, which are quantitatively validated by making comparisons with reported results in the literature dealing with the same dataset. By integrating the Transformer encoder with an unsupervised denoising framework, we showcase the benefits of learning a well-suited wavelet transform at each level during the decomposition process and apply a learnable hard-thresholding method to effectively evaluate noise in raw vibration data. This, combined with the self-attention mechanism in the Transformer architecture, enables us to leverage attention-based and residual temporal data processing, capturing time-varying relationships from segmented samples of raw signals.

Our method enables the use of vibration data as input to a deep-learning architecture, a scenario typically avoided in the literature due to challenges in constructing effective designs resilient to variations in input durations. Additionally, our approach generates diagnostic information directly from the waveform patterns of the vibration data, potentially enhancing the models’ ability to analyze and interpret the data.

In our future work, we plan to extend the proposed mixed model to handle more complex data scenarios, such as longer sample data or data with irregular sampling. We see opportunities to enhance the model through advanced hyperparameter-tuning techniques. An ablation analysis to better understand the contributions of network components may be another direction of future work.

## Figures and Tables

**Table 1:** Characteristics of experiments from IMS bearing dataset.

Properties	Values
Sampling Frequency	20,480 Hz
Operating Speed	2000 RPM
Static Loading	26.7 kN
Bore Diameter	49.2 mm
Max Runtime	34 days 12 h

**Table 2:** Characteristics of experiments from CWRU bearing dataset.

Features	Content
Test bench	Motor with 2 HP power
	Torque transducer
	Dynamometer
	Control electronics
Diameters of defects in inches (millimeters)	0.007 inches (0.178 mm)
	0.014 (0.356)
	0.021 (0.533)
Telemetry measurements	Drive end (DE)
	Fan end (FE)
	Base (BA)
Conditions	1 HP load applied to the motor
	Shaft rotating speed of 1772 rpm
	48 kHz sampling frequency of the accelerometers
Parts of the bearing	Ball
	Inner race
	Outer race

**Table 3:** Model evaluation 10 classes.

	Precision	Recall	F1-Score	Support
0	1.00	0.97	0.98	32
1	0.95	1.00	0.97	39
2	1.00	1.00	1.00	38
3	1.00	0.97	0.98	30
4	1.00	1.00	1.00	30
5	1.00	1.00	1.00	86
6	1.00	1.00	1.00	35
7	1.00	1.00	1.00	38
8	1.00	0.97	0.98	30
9	0.98	1.00	0.99	41
Accuracy			0.99	399
Macro avg	0.99	0.99	0.99	399
Weighted avg	0.99	0.99	0.99	399
Test accuracy	0.9949874686716792			

**Table 4:** Model evaluation 4 classes.

	Precision	Recall	F1-Score	Support
0	1.00	1.00	1.00	32
1	0.99	0.99	0.99	107
2	1.00	1.00	1.00	151
3	0.99	0.99	0.99	109
Accuracy			0.99	399
Macro avg	1.00	1.00	1.00	399
Weighted avg	0.99	0.99	0.99	399
Test accuracy	0.974937343358396			

**Table 5:** Model’s performance comparison and remarks.

Ref.	Results	Remarks
Our model	99.5% and 97.5% test accuracy for validation on dataset CWRU with 10-class and 4-class classification, respectively.	Various evaluation metrics are utilized. The model is trained and validated using fixed-window-length data. Despite the training and inference processes each taking several seconds for a sample size of 1024, the model maintains high performance. Incorporating an attention mechanism may enhance explainability.
[18]	Model evaluation has 96% accuracy in the test set, 97.96% of F1-score. Performance is verified with various imbalance ratios and parameters when transforming data.	Injecting noise using GAN is tricky when the noise ratio and distribution need to be carefully managed to ensure accuracy and effectiveness.
[19]	99.73% training accuracy for the chosen dataset.	Performance depends on the wavelet family and the number of segmentation samples from the original dataset. The complexity of the assembled model needs to be considered.
[21]	100% training accuracy in all the classes.	Because of the inherent intricacy of the hidden layers, it is challenging to understand how the learnt model works. The data samples are selected and reconstructed from the original data, in which each sample has the same time course.
[22]	With 8192 samples, the model’s accuracy was 99.34 percent.	When the sample size is 8192, the training process takes about 40 min on average.
[27]	99.7% accuracy.	Each fault data sample contains the same number of data points, and the sample length is fixed.
[23]	98.47% accuracy in testing.	The number of viable options for building child models is too great, and the research’s scope is too broad. Despite the fact that random actions are generated to prevent local optimal solutions, it is still easy to get stuck in the local ideal solution.

## Data Availability

The data presented in this study were derived from the following resources available in the public domain: [IMS Bearing Dataset, NASA Prognostics Data Repository, https://www.nasa.gov/intelligent-systems-division/discovery-and-systems-health/pcoe/pcoe-data-set-repository (accessed on 2 May 2022)] and [CWRU Bearing Data Center, https://engineering.case.edu/bearingdatacenter/download-data-file (accessed on 2 May 2022)].
